# Effect of Platelet Lysate on Human Cells Involved in Different Phases of Wound Healing

**DOI:** 10.1371/journal.pone.0084753

**Published:** 2013-12-27

**Authors:** Maria Chiara Barsotti, Paola Losi, Enrica Briganti, Elena Sanguinetti, Angela Magera, Tamer Al Kayal, Roberto Feriani, Rossella Di Stefano, Giorgio Soldani

**Affiliations:** 1 Department of Surgery, Medical, Molecular, and Critical Area Pathology, University of Pisa, Pisa, Italy; 2 Institute of Clinical Physiology, National Research Council, Massa, Italy; 3 Kedrion SpA, Castelvecchio Pascoli, Italy; University Hospital Hamburg-Eppendorf, Germany

## Abstract

**Background:**

Platelets are rich in mediators able to positively affect cell activity in wound healing. Aim of this study was to characterize the effect of different concentrations of human pooled allogeneic platelet lysate on human cells involved in the different phases of wound healing (inflammatory phase, angiogenesis, extracellular matrix secretion and epithelialization).

**Methodology/Principal Findings:**

Platelet lysate effect was studied on endothelial cells, monocytes, fibroblasts and keratinocytes, in terms of viability and proliferation, migration, angiogenesis, tissue repair pathway activation (ERK1/2) and inflammatory response evaluation (NFκB). Results were compared both with basal medium and with a positive control containing serum and growth factors. Platelet lysate induced viability and proliferation at the highest concentrations tested (10% and 20% v/v). Whereas both platelet lysate concentrations increased cell migration, only 20% platelet lysate was able to significantly promote angiogenic activity (p<0.05 vs. control), comparably to the positive control. Both platelet lysate concentrations activated important inflammatory pathways such as ERK1/2 and NFκB with the same early kinetics, whereas the effect was different for later time-points.

**Conclusion/Significance:**

These data suggest the possibility of using allogeneic platelet lysate as both an alternative to growth factors commonly used for cell culture and as a tool for clinical regenerative application for wound healing.

## Introduction

Wound healing is a dynamic process highly dependent on the coordinated functions of inflammatory cells, endothelial cells, fibroblasts, and keratinocytes, with the ultimate goal of restoring skin integrity [1]. This process is a complex integration of cascades, which requires multiple cytokines and growth factors for different stimulatory and inhibitory functions to initiate and direct different phases. Factors involved in wound healing include platelet-derived growth factor (PDGF), transforming growth factor (TGF) and vascular endothelial growth factors (VEGF). Recombinant growth factors advent was followed by their premature and empiric introduction into clinical practice for wound healing treatment, in order to reproduce the physiological process. However, few of them are available for clinical use and they have practical limitations due to their short half-life and excessive cost. The limited success and heterogeneous clinical results obtained by the use of single growth factors are probably related to the requirements for multiple signals to achieve a complete regeneration process, assuming that no single exogenous agent can effectively mediate all aspects needed for tissue repair [2]. Thus, delivery of a wide range of biological mediators is probably required for efficient and complete healing, recreating and propagating the normal sequence of events. 

Platelets play a critical role in wound healing, actively promoting cell recruitment, tissue regeneration and matrix remodelling [3], angiogenesis and blood vessel maturation [4]. These effects are largely due to the high content of bioactive molecules such as growth factors and mediators, released by activated platelets during blood coagulation [5]. The increased understanding of the physiological roles of platelets in wound healing after tissue injury led to the idea of using platelets, the natural source of growth factors, as therapeutic tools. Local application of concentrated platelet preparations might achieve a complex of growth factors which stimulates the healing process in a more physiological way. 

The clinical use of platelets for wound healing applications is nowadays restricted to autologous platelet gels. The efficacy of topical platelet gel application has been evaluated in both animal and clinical studies, observing a significant improvement in wound healing [6]. However, the process of obtaining autologous platelet gel is difficult to standardize and impractical for extensive use. In fact, platelet profiles and effects change in relation to the properties of original blood samples and methods of fabrication [7]. Although non-homogeneous results have been reported, several studies regarding the use of platelet concentrates or gels showed reduced inflammation, improved bone regeneration and improved hard and soft tissue wound healing [6]. In addition to the treatment of chronic wounds and ulcers, platelet derivatives and platelet-released growth factors find application also in orthodontics, dental surgery and plastic surgery [2]. 

Another platelet derivative with potential clinical application is platelet lysate (PL). PL, obtained by repeated freezing-thawing of platelet enriched blood samples, contains a cocktail of immediately available growth factors and cytokines that can promote tissue regeneration. Human PL has been recently proposed as an alternative to fetal bovine serum (FBS) for clinical-grade cell expansion for regenerative medicine [8,9], as FBS carries the risk of xenogenic immune reactions and of transmitting viral and prion diseases. PL was shown to be superior to or at least the same compared to FBS in most studies regarding stem cells [8] and bone marrow mesenchymal cells expanded in medium supplemented with PL instead of FBS have been used in clinical trials [10]. However, PL donor age affects proliferation, senescence and osteogenic differentiation of MSCs [11].

The aim of this study was to further characterize the wound healing properties of increasing concentrations of human allogeneic pooled PL on cells involved in the different phases of wound healing. In particular, the effect of increasing concentrations of PL was studied on human cell types such as monocytes, endothelial cells, fibroblasts and keratinocytes. Further analyses on the activation of intracellular pathways involved in both angiogenesis and tissue repair were performed with selected PL concentrations. PL at the two highest concentrations tested was able to increase in a similar way cell viability, proliferation and migration of different cell populations. On the other way, a differential effect of these two concentrations was observed regarding angiogenesis and intracellular pathway activation. 

## Materials and Methods

### Ethics statement

Transfusion units of platelet rich concentrates, derived from buffy coats, were obtained from voluntary healthy blood donors at Blood Transfusion Center "SS. Giacomo e Cristoforo Hospital", Massa, Italy (www.usl1.toscana.it), a structure that has been accredited by Regional Health Authority to collect whole blood and prepare donor-derived platelet concentrates. The approval from institutional review board or ethics committee was thus not necessary. Written informed consent from the blood donors was obtained for using platelet rich concentrates for research. 

### Platelet lysate preparation

Whole blood was separated into platelet-rich plasma (PRP) and erythrocytes by centrifugation (300 g for 10 min). PRP was then centrifuged at 1500 g for 15 min to obtain the platelet pellet. After washing, the pellet was gently resuspended in NaCl (0.9% w/v) saline solution and concentrated to 1.3x10^9^ platelets/ml. Finally, to obtain PL, the platelet concentrate was frozen (-80°C) and thawed (37°C) three times and stored in aliquots at -80°C until use. PL samples were vortexed for 1 min to obtain a homogeneous suspension before use. Pooled samples prepared from PRP obtained from five different donors were used to minimize variations between donors. 

### Pro-angiogenic growth factor quantification in platelet lysate

The levels of PDGF-AB, TGF-β1 and VEGF were assessed in PL samples using enzyme-linked immunosorbent assays for these factors (Quantikine, R&D Systems, Inc., Minneapolis, MN, USA), according to manufacturer’s instructions. Samples for TGF-β1 analysis were acid activated with 1 N HCl. Growth factor concentrations were measured in triplicate in three different samples.

### Cell isolation and culture

PL biological effect on cell viability, proliferation, migration, angiogenesis, intracellular pathways activation and inflammatory response was performed using four different human cell types involved in the different phases of wound healing: monocytes, endothelial cells, fibroblasts and keratinocytes. 

THP-1 (human acute monocytic leukemia cell line, ICLC HTL97014) were maintained at 2–9x10^5^ cells/ml cell density in RPMI 1640 supplemented with 10% FBS, 2 mM L-Glutamine, 100 μg/ml streptomycin and 100 U/ml penicillin. Each experiment was performed on cells that demonstrated viability rates >95% after staining with Trypan blue dye for dead cell exclusion.

Human umbilical vein endothelial cells (HUVECs) were obtained by treatment with 0.1% collagenase type II (Worthington Biochemical Corporation, Lakewood, NJ, USA) and 0.5% albumin solution to allow endothelial cell detachment from the vessel wall [11]. Isolated cells were cultured in Medium 199, supplemented with 20% FBS, 2mM L-Glutamine, 100 μg/ml streptomycin, 100 U/ml penicillin, 100 μg/ml heparin from porcine intestinal mucosa and 50 μg/ml epithelial growth factor (EGF). Experiments were performed at passage 3, using a pool from three different donors.

Human dermal fibroblasts HFFF2 (ICLC HL97002) and dermal keratinocytes NCTC 2544 (ICLC HL95002) were cultured in DMEM supplemented with 10% FBS, 2 mM L-Glutamine, 100 μg/ml streptomycin and 100 U/ml penicillin.

Medium was routinely changed every 3 days and at confluence cells were subcultured (split ratio 1:3) by trypsinization (0.5% trypsin/0.02% EDTA).

The culture media described for each cell type were defined as complete media. Experiments on PL effects were performed in absence of serum and EGF.

All the cell cultures were incubated at 37°C in a humidified atmosphere with 5% CO_2_.

Culture media, reagents and serum were purchased from Sigma-Aldrich (Saint Louis, MO, USA).

### Cell viability assay

Cell viability was assessed using the XTT colorimetric assay (Sigma-Aldrich) based on the reduction of sodium 2,3-bis(2-methoxy-4-nitro-5-sulfophenyl)-2H-tetrazolium-5-carboxanilide inner salt (XTT), to an orange soluble formazan by the succinate dehydrogenase system of the mitochondrial respiratory chain. The reaction requires the presence of an electron coupling reagent, N-methyldibenzopyrazine methyl sulfate (PMS), serving as an intermediate electron acceptor. Only viable cells with active mitochondria reduce significant amounts of XTT to formazan. 

HUVECs were seeded into pregelatinized 96-well plates at a density of 8×10^3^ cells per well. After 24 hours of incubation, when cell growth was in logarithmic phase, the medium was carefully decanted and replaced with 200 μl/well of serum-free medium containing 1, 5, 10 or 20% (v/v) PL. Complete medium was used as a reference. After a 48-hours exposure, the viable cell number was determined by XTT dye reduction. A XTT (1 mg/ml) and PMS (0.025 mM) labelling mixture was added to each well and incubated for 4 hours at 37°C. At the end of incubation time, the optical density (OD) was measured at 450 nm wavelength using a microplate reader (Spectrafluor Plus; TECAN Austria GmbH, Grödig, Austria). The percentage of cell viability was calculated vs. the complete medium (assumed as 100%). 

### Cell proliferation assay

Cell proliferation was evaluated by 5-bromo-2'-deoxyuridine (BrdU) assay. BrdU is incorporated instead of thymidine into the DNA of new synthesis, so BrdU incorporation is a suitable method for the quantitative measurement of cell proliferation. 

HUVECs were seeded onto pregelatinized 96-well plates at a density of 5x10^3^ cells/well for 24 hours. After that, when cell growth was in the logarithmic phase, the medium was carefully decanted and replaced with 200 μl/well of medium containing PL (1, 5, 10 or 20%). Complete medium was used as a reference. After 48 hours of PL incubation, cell proliferation was assessed by BrdU assay (Roche Diagnostics, Mannheim, Germany). BrdU reagent was added to each well and after 4 hours of incubation the assay was performed following the procedure prescription. The OD was measured at 450 nm wavelength using the microplate reader. The percentage of cell proliferation was calculated vs. the complete medium (assumed as 100%). 

### Monocyte chemotaxis assay

Monocyte chemotaxis was measured using a 6.5mm Transwell® with 5.0 µm pore polycarbonate membrane insert (Corning Costar, Cambridge, MA, USA). THP-1 cells were resuspended at 5x10^5^ cells/ml and 200 µl of cell suspension were transferred to the upper chamber of each transwell. The lower chamber was filled with 400 µl of medium containing PL at different concentrations (5, 10 or 20%). For control wells, serum-free medium and Monocyte Chemotactic Protein-1 (MCP-1, 10 ng/ml) enriched medium were used as chemotactic negative and positive control, respectively. Recombinant human MCP-1 was obtained from Invitrogen Corp. (Carlsbad, CA, USA).

THP-1 cells were allowed to transmigrate for 120 minutes in humidified atmosphere with 5% CO_2_. Cells migrated through the polycarbonate filter pores in response to chemoattractant stimuli were then counted by Bürker chamber under light microscopy.

### Scratch closure assay

PL effect on wound healing-associated migration was assessed employing scratch-wounded keratinocyte and fibroblast monolayer models [5,12]. Briefly, 8x10^4^ keratinocytes NCTC 2544 and 5x10^4^ fibroblasts HFFF2 were seeded into 24-well plates, cultured to confluence and scratched by scraping with a 0.1–10 µL pipette tip. Following PBS washes, cultures were re-fed with 500 μl/well of PL diluted at different concentration (5, 10 and 20%) in serum-free culture medium. Control wells received serum-free medium or complete culture medium. 

At 0, 6, 18, 24, 48 and 72 hours after scratching, digital images of cells were captured by a phase contrast microscope (Axiovert 25, Zeiss, Milan, Italy; O.M. 50X) equipped with a digital camera (EOS 1000D, Canon, Milano, Italy). Scratch closure was qualitatively analyzed at each time-point with respect to time 0. For a quantitative evaluation, scratch closure was determined as the difference between scratch width at time 0 and each time-point. Each well was marked below the plate surface by drawing a vertical line, to allow identification of the same scratched area in order to take consistent pictures. Scratch area was measured using Image J software (NCBI). Migration rate was expressed as percentage of scratch closure compared to the initial area and was calculated as follows = [(At0 -At)/At0]*100, where At0 is the scratch area at time 0, and At is the correspondent scratch area at 6, 18, 24, 48 or 72 hours. The values shown are the means of three wells from three independent experiments.

### *In vitro* angiogenesis assay

To assess the potential effect of PL on *in vitro* angiogenesis, HUVECs were treated with PL for 48 hours before being detached and seeded on Matrigel.

Briefly, HUVECs (7x10^4^ cell/well) were seeded in complete medium on gelatin-coated 6-well plates. At subconfluence, medium was replaced with serum-free medium in absence (0% PL) or in presence of PL (10% and 20%). Complete medium was used as a reference. After 48 hours of incubation at 37°C, 5% CO_2_, cells were detached and their number and viability was assessed by Trypan Blue staining. 7x10^4^ cells/well were then seeded on 24-well plates pre-coated with 300 μl of 10 mg/ml Matrigel (BD Biosciences, San Jose, CA, US), thawed on ice at 4°C, and allowed to solidify at 37°C for 30 min before cell seeding [13]. After 4 and 8 hours of incubation at 37°C, tubules were observed under an inverted microscope (Hund, Wetzlar, Germany) equipped with a digital camera (Nikon, Sesto Fiorentino, Italy). Pictures (n=10, 10X magnification) captured after 8 hours were analyzed by the image analysis software ImageJ (public domain, Image Processing and Analysis in Java, National Institutes of Health), with the plug-in AngioJ for angiogenesis assay. Morphometric parameters (area %, tubule total length and number of branching points) were calculated versus the complete medium (assumed as 100%). 

### Activation of intracellular pathways and inflammatory response

Extracellular-signal-regulated protein kinase (ERK1/2) and nuclear factor κB (NFκB) activation was detected by specific commercial immunoenzymatic assay (Fast Activated Cell-based ELISA, FACE, Active Motif, Carlsbad, CA, USA), enabling monitoring of the levels of proteins activated by phosphorylation. Briefly, HUVECs were seeded on 96-well plates and treated with 0, 10 or 20% PL in serum-free medium for 15, 30 and 60 minutes (ERK1/2) or 1, 24 and 48 hours (NFκB). Complete medium, containing both EGF and 20% FBS, was used as a positive control. Next, cells were fixed and anti-phospho-protein or anti-total protein (primary antibodies) were added into the wells and incubated. After washing, an anti-mouse IgG conjugated to horseradish peroxidase (HRP) (secondary antibody) was added to the wells for 1 hour. A TMB (3,3',5,5' tetramethyl-benzidine) substrate solution was added to the wells and color developed in proportion to the amount of protein. Absorbance values (OD_450_) were normalized for the cell number in each well, using the Crystal Violet 0.1% solution assay (OD_595_). The values of total and phosphorylated proteins were calculated by the value normalization to the ratio OD_450_/OD_595_ of control cells and plotted. 

### Statistical analysis

Each test was conducted at least three separate times. Results are expressed as mean ± S.D. of six replicate wells for each samples and controls.

Data were analyzed by StatView^TM^ 5.0 software (SAS Institute, Cary, NC, USA). The means were statistically compared by the independent Student’s *t*-test. Values of p<0.05 were considered statistically significant.

## Results

### Pro-angiogenic growth factor quantification in platelet lysate

Mean content (± SD) of PDGF-AB, TGF-β1 and VEGF measured by ELISA assay in PL samples were respectively 42 ± 9 ng/ml, 64± 5 ng/ml and 740 ± 110 pg/ml. These concentrations are approximately equivalent to a growth factor content of 8.4 ng/ml (PDGF-AB), 12.8 ng/ml (TGF-β1) and 148 pg/ml (VEGF), for cell treatment with 20% v/v PL.

### Cell viability assay

**Figure 1:**
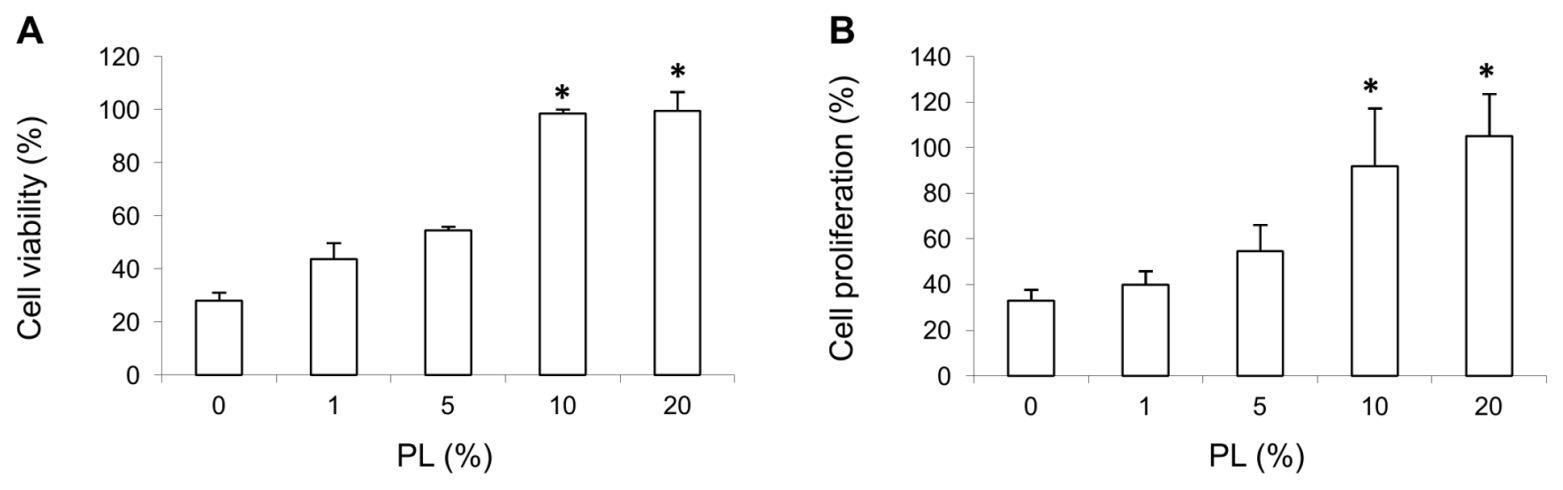
HUVEC viability and proliferation. HUVEC viability (**A**) and proliferation (**B**) were assessed by XTT assay and BrdU incorporation assay, respectively, following 48 hours of incubation with different PL concentrations (1, 5, 10 and 20%). The percentage of cell viability and proliferation were calculated versus the complete medium (assumed as 100%). Data are means ± SD of values obtained from three independent experiments with six replicates each. * p<0.01 vs. serum-free medium, 1 and 5% PL. .

Cell viability of HUVECs exposed to increasing PL concentrations for 48 hours was evaluated by XTT assay. HUVEC viability as compared to the complete control medium (assumed as 100%) is shown in Figure 1A. HUVECs incubated in serum-free medium showed a PL dose-related increase in mitochondrial metabolism. In particular, the removal of serum and EGF from culture medium reduced the cell viability to 28% (0% PL), with no significant variation by adding 1 or 5% PL. A significant increase in cell viability (p<0.01 vs. 0% PL) was observed with higher concentrations of PL (10 and 20%), comparable to the complete medium.

### Cell proliferation assay

Cell proliferation of HUVECs exposed to increasing PL concentrations for 48 hours under serum-free conditions was determined by BrdU assay. DNA synthesis as compared to the complete culture medium (assumed as 100%) is shown in Figure 1B. The 1 and 5% PL were not significantly different from the negative control (0% PL), whereas 10 and 20% PL induced a significant increase in cell proliferation(p<0.01 vs. 0% PL), comparable to complete medium. 

### Monocyte chemotaxis assay

**Figure 2:**
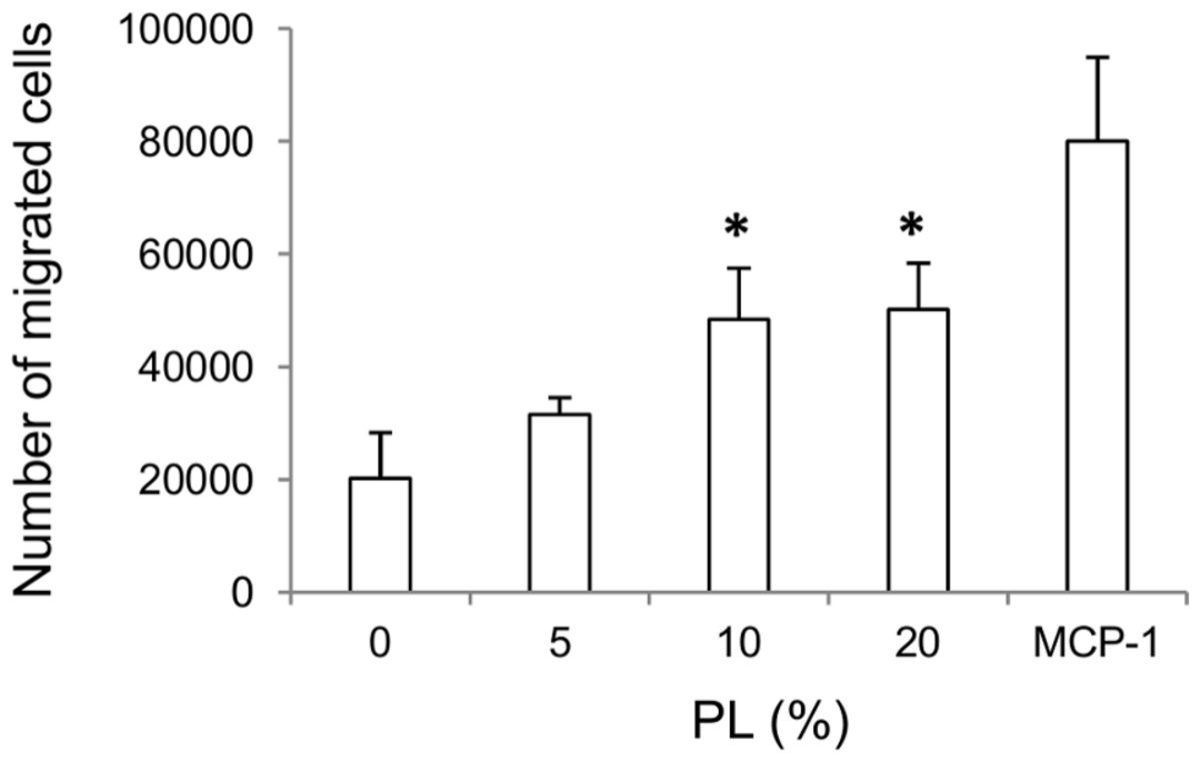
Monocyte chemotaxis assay. The chemotactic effect of PL on monocytes was assessed following 2 hours of cell migration towards different PL concentrations (5, 10 and 20%). Positive and negative controls are represented by 10 ng/ml MCP-1 and by serum-free medium, respectively. Data (n=3) are means ± SD of six replicates. * p<0.05 vs. both serum-free medium and 5% PL. .

PL effect on cell migration rate was assessed by a chemotaxis assay, using the chemoattractive protein MCP-1 as a positive control (Figure 2). Both 10 and 20% PL produced a comparable significant increase (p<0.05) in migrating cell number as compared to both serum-free condition and 5% PL. 

### Scratch closure assay

PL effect on cell migration was determined by scratch assay with dermal keratinocytes (NCTC 2544) and fibroblasts (HFFF2), in order to reproduce cell spreading across the wound to form a new epithelial layer. 

**Figure 3:**
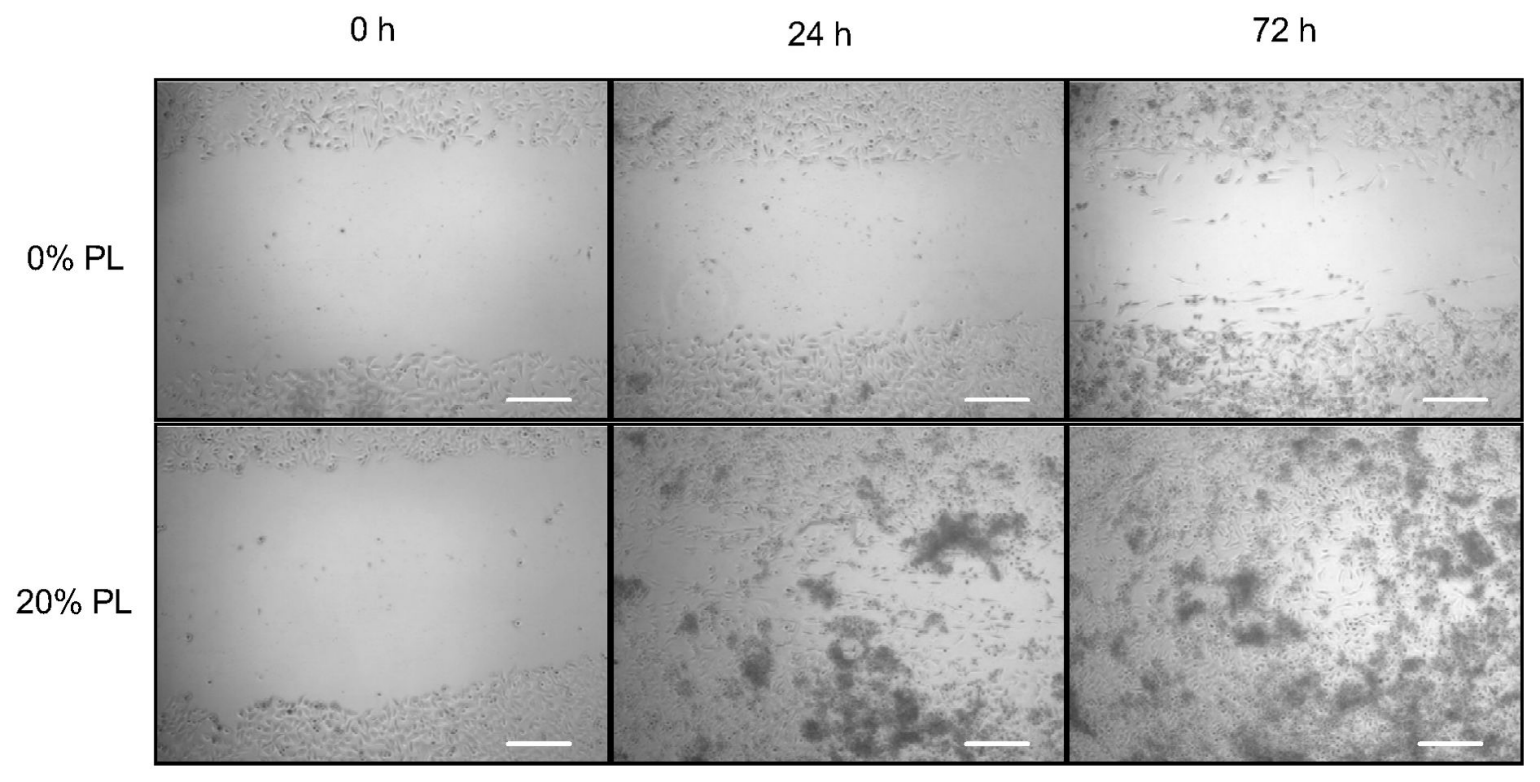
Scratch closure assay. Representative phase contrast micrographs of 0 and 20% PL-treated keratinocytes at 0, 24 and 72 hours are shown. Scale bar: 200 μm.

**Figure 4:**
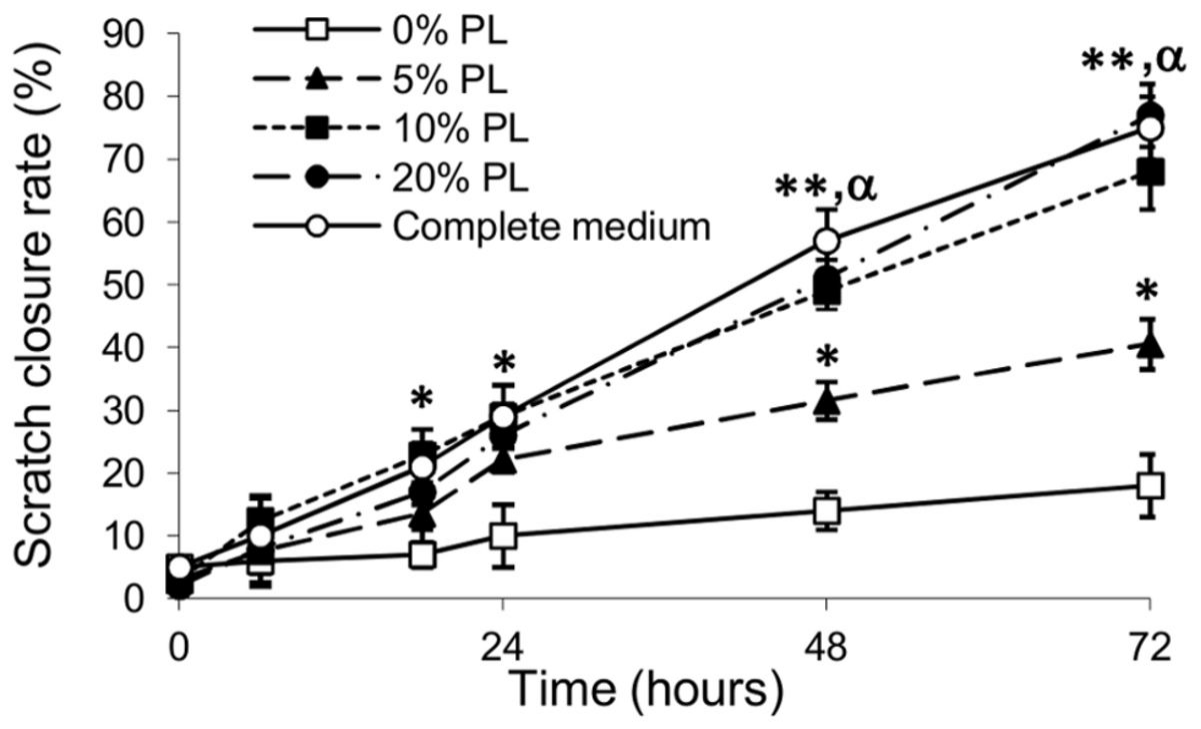
Scratch closure rate. Quantification of the effect of PL on scratch closure. A progressive closure was observed with PL treatment, starting from 18 hours. Scratch wounded cells incubated with 10% and 20% PL for 48 and 72 hours showed a significant higher scratch closure as compared to 5% PL, comparable to complete medium (positive control). *: p<0.05; **: p<0.005, all vs. 0% PL. α: p<0.05 vs. 5% PL. Bars represent the means ± SD of values obtained from three independent experiments each one with three replicates per group of treatment. Closure rates are expressed as percentage of scratch closure after 6, 18, 24, 48 and 72 hours compared to the initial area.

Figure 3 shows representative photomicrographs of keratinocytes, scratch wounded and exposed to serum-free medium (0% PL) and 20% PL, at 0, 24 and 72 hours. A significant effect on scratch closure was observed with all PL concentrations as compared to 0% PL, starting from 18 hours (Figure 4). Scratch wounded cells incubated with 10% and 20% PL for 48 and 72 hours showed a significant higher scratch closure as compared to 5% PL, comparable to complete medium (positive control). No significant difference was observed between 10 and 20% PL treatment at each time-point. 

Incubation of fibroblasts with different PL concentration induced a similar migration rate (data not shown). 

### *In vitro* angiogenesis assay

**Figure 5:**
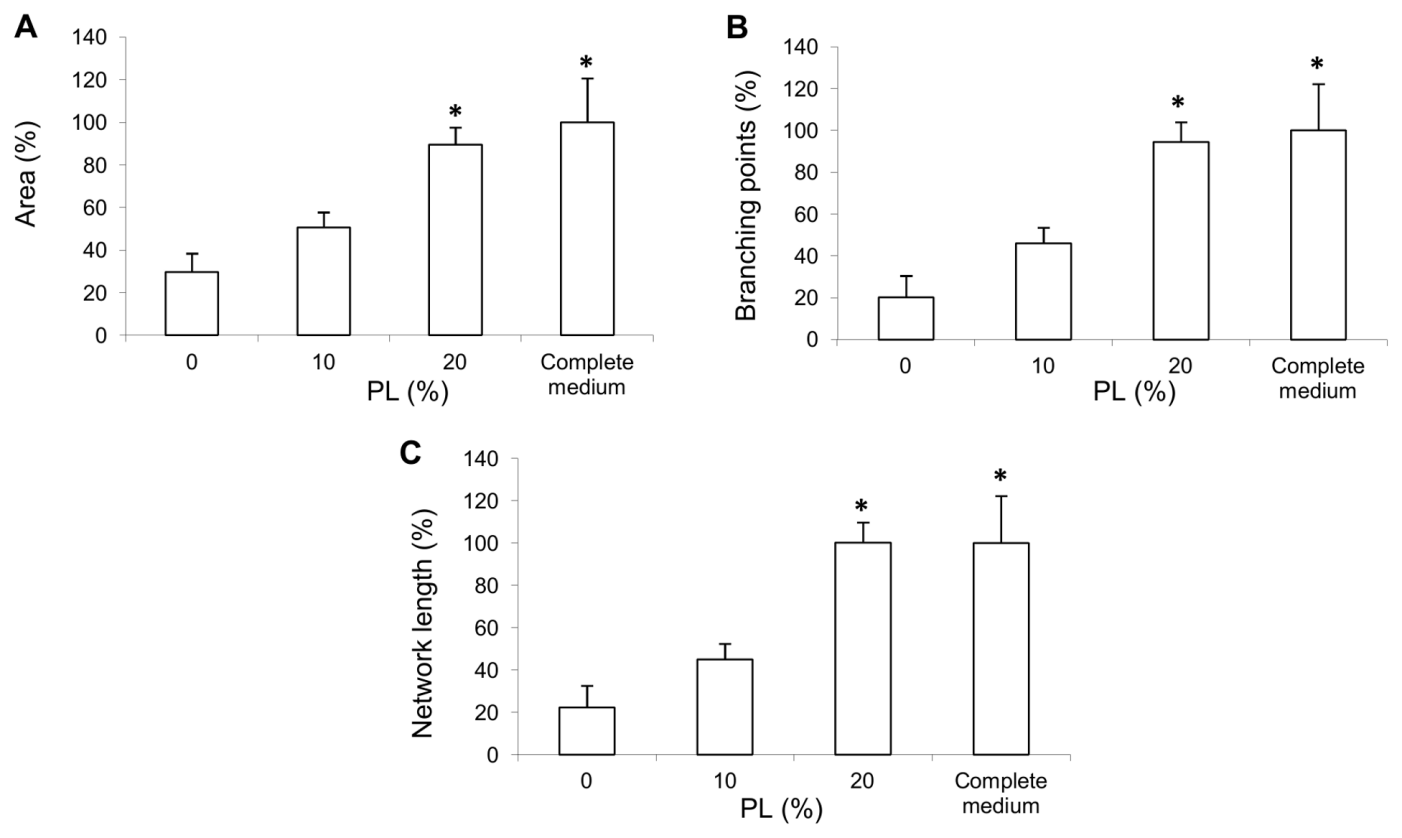
Angiogenesis assay. The effect on HUVECs of 10% or 20% PL-pre-treatment in serum-free medium for 48 hours, followed by seeding on Matrigel, is shown. The following morphometric parameters were evaluated after 8 hours of angiogenesis assay by AngioJ analysis: area % (**A**), branching points (**B**), total tubule length (**C**). Data are expressed as mean±SD of three independent experiments carried out in duplicate. The 20% PL significantly increased morphometric parameters (p<0.05 vs. 0% PL) comparably to the complete medium. A non significant increase was observed with 10% PL.

The angiogenic capability of human endothelial cells after PL pre-treatment was evaluated after 8 hours on matrigel (Figure 5). HUVEC preincubation in serum-free medium (0% PL) significantly decreased angiogenic capability as compared to the complete medium. Cells pre-incubated with 20% PL exhibited the highest tubule-forming capability in terms of morphometric parameters (area %, Figure 5A; number of branching points, Figure 5B; total tubule length, Figure 5C), which was comparable to those of cells incubated with complete medium. A non significant increase in morphometric parameters was observed with 10% PL, as compared to 0% PL.

### Activation of intracellular pathways and inflammatory response

**Figure 6:**
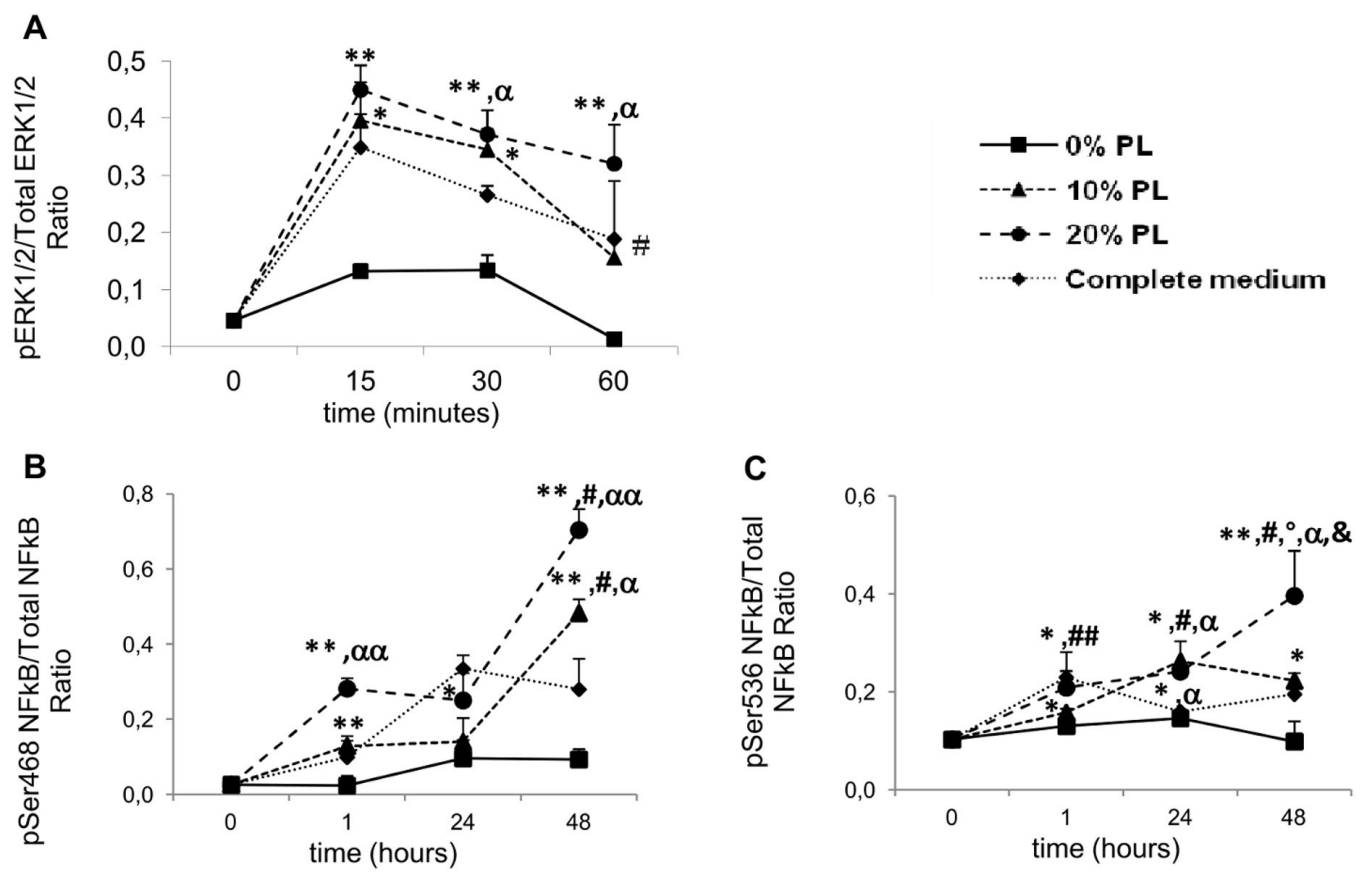
Activation of intracellular pathways and inflammatory response. Time-course of ERK1/2 and NFκB phosphorylation after 10% or 20% PL treatment of HUVECs. Data are expressed as mean±SD of the ratio between the absorbance referred to the phosphorylated form and to the corresponding total protein. (**A**) Phosphorylation of ERK1/2. *: p<0.05; **: p<0.005, all vs. 0% PL. #: p<0.05 vs. both 15 and 30 min. α: p<0.001 vs. complete medium. (**B**) Phosphorylation of NFκB at Ser 468. *: p<0.05; **: p<0.01, all vs. 0% PL. #: p<0.005 vs. both 1 and 24 hours. α: p<0.05; αα: p<0.01, all vs. complete medium. (**C**) Phosphorylation of NFκB at Ser 536. *: p<0.05; **: p<0.0001, all vs. 0%PL. &: p<0.05 vs. 10% PL. #: p<0.05; ##: p<0.005, all vs. 1 hour. °: p<0.01 vs. 24 hours. α: p<0.05 vs. complete medium.

10% PL induced ERK1/2 activation in HUVECs starting from 15 min (p<0.05 vs. 0% PL), with a constant value at 30 min (p<0.05 vs. 0% PL) and a decrease at 60 min (p<0.05 vs. 15 and 30 min); 20% PL effect was comparable (p<0.005 vs. 0% PL), except at 60 min, when the decrease was much less marked (p<0.05 vs. 15 min) (Figure 6A). The effect was comparable to the positive control at 15 min for both PL concentrations, while at 30 min ERK1/2 activation was significantly higher for 20% PL (p<0.001 vs. positive control). 

Regarding NFκB, two major phosphorylation sites (Ser 468 and Ser 536) of p65 subunit were evaluated. As a whole, PL activated NFκB at both phosphorylation sites. At Ser468 (Figure 6B), although the trend was similar for both PL concentrations, 10% PL significantly induced activation only at 1 and 48 hours (p<0.01 vs. 0% PL ), while 20% PL effect on activation was significant at every time-point (1 hour: p<0.01; 24 hours: p<0.05; 48 hours: p<0.01 all vs. 0% PL). At 48 hours, activation was significantly increased compared to the former time-points for both PL concentrations (p<0.005). At Ser536 (Figure 6C), the effect of the two concentrations was comparable at 1 and 24 hours (p<0.05 vs. 0% PL), diverging at 48 hours (10% PL: p<0.05; 20% PL: p<0.0001, all vs. 0% PL; 20% PL: p<0.01 vs. 24 hours), with a significant increase in activation for 20% PL (p<0.05 vs. 10% PL). Activation was also higher as compared to the complete medium (p<0.05).

## Discussion

Wound healing is a complex and dynamic process including inflammation, proliferation and remodeling, with crucial signaling pathways. It is generally accepted that growth factors and cytokines have an essential role as regulators of basic cell functions [2]. The role of human platelet growth factors in different stages of the wound healing cascade has been widely demonstrated. Thus, PDGF delivered in a topical aqueous-based gel (Regranex, Ethicon, Inc., Irvine, CA) has received FDA approval for treatment of limb diabetic ulcers [14] and TGF-β has been tested in several clinical studies [15]. However, the use of single growth factors has shown limited success and heterogeneous clinical results [2], encouraging the use of a combination of biological mediators, as represented by platelet derivatives, for efficient tissue regeneration. 

PL, obtained by freezing/thawing platelets, has been shown to deliver more growth factors compared with PRP [16]. However, it is essential to standardize platelet derivatives to evaluate the effectiveness of treatments. The complexity of establishing parameters is linked to variability and bioavailability of mediators in platelets and to variability in product preparation. Recently, the content of 1×10^9^ platelets/ml has been proposed as a reference realistic parameter [17]. The allogeneic product that we used for this study matched this parameter as platelet content was 1.3×10^9^/ml. In view of clinical setting, it is preferable that PL is manufactured according to the same strict regulations as practiced in the blood processing departments of blood banks and the general hospital environment [18]. PL was obtained from healthy donors, offering an advantage over patient derivatives. A pool of donors was used to minimize individual variability. We evaluated the concentration of growth factors playing a key role in wound healing because of their effect on different cellular activities and biological responses [19-21]. PDGF-AB, TGF-β1 and VEGF amounts resulted in the same order of size of previous studies on PL [16,22]. Skin wound healing requires recruitment or activity of different cell types involved in different stages of this process, such as keratinocytes, endothelial cells, fibroblasts and inflammatory cells. In the present study, we evaluated the *in vitro* effect of different concentrations of PL, ranging from 1% to 20%, on proliferation and activity of such cell types. Unlike previous studies that were performed adding PL to FBS-containing media [12,23], we evaluated PL effects in absence of serum and growth factors. Cell viability and proliferation, evaluated by XTT and BrdU assay, and cell migration induction, determined by monocyte chemotaxis and scratch wound experiments, revealed that PL at 10% and 20% promotes cell activity, accelerating *in vitro* wound healing of various cell types. The results we obtained on viability and activities of different human adult cells may support the use of PL as a substitute for both FBS and growth factors. However, PL concentration, must be accurately chosen, as its effects are dose-related.

Platelets play a critical role in regulating angiogenesis in wound healing. As platelet alpha granules contain several growth factors and chemokines that positively and negatively regulate angiogenesis, a spatial-temporal control of the release of angiogenic regulators to allow for a balance of anti- and pro-angiogenic factors is required [24]. 

Although the angiogenic effect of single platelet factors has been extensively studied, there are few data available on the angiogenic effect of platelet derivatives as PL. Stabilized freeze-dried platelets were able to induce tubule formation as the freeze-drying process preserved growth factors essential for wound healing, such as PDGF-BB and TGF-β1 in quantities similar to fresh platelets [25]. The differential release of angiogenesis stimulators and inhibitors from platelets was also studied, showing that activation with adenosine diphosphate stimulated the release of VEGF but not endostatin, promoting capillary tube formation [26]. A very recent study evaluated platelet-stimulated angiogenesis focusing mainly on the release of angiogenesis-regulating factors from their α-granules upon aggregation [27].

In our study, we treated endothelial cells with the higher PL concentrations (10% and 20%), demonstrating that PL affects angiogenesis, as shown by matrigel *in vitro* assay. Interestingly, while both 10% and 20% PL significantly increased cell viability and proliferation, they did not have the same effect on angiogenesis, as only 20% PL, corresponding to a physiological platelet concentration, showed a significant promoting effect. The non-significant effect of 10% PL could be explained by a lower amount of pro-angiogenic factors and/or by a different regulation of the pathways involved in the angiogenesis process. 

Another advantage of the use of platelet derivatives has been proposed recently. Platelet-derived preparations might accelerate regeneration of difficult-to-heal wounds by triggering an inflammatory cascade and playing an antimicrobial role [28,29]. For this reason, we evaluated in endothelial cells the activation of important inflammatory pathways involved in both angiogenesis and tissue repair such as ERK1/2 and NFκB, performing a quantitative and highly sensitive non-radioactive assay that does not require cell extraction. ERK1/2 signaling cascade activates many nuclear transcription factors, mediating response to different growth factors such as VEGF to coordinate and regulate multiple cell functions, initiating a complex and integrated network of signaling pathways [30]. ERK1/2 is crucial for cutaneous wound healing [31], as it is itself activated in response to injury and phosphorylated by growth factors involved in this process, such the ones produced by platelets [32]. The transcription factor NFκB, a key component for the expression of many cellular genes, has been implicated in the signaling pathways of several growth factors produced by platelets, controlling cytokine expression and regulating inflammatory response, with effects on corneal wound healing and angiogenesis [33]. Gene silencing of NFκB was shown to impair *in vitro* wound healing by keratinocytes [34]; moreover, natural products also used for wound healing, were able to exert potent antiproliferative and antiangiogenic activity by blocking the NFκB cascade in different cell types, including endothelial cells [35]. The interconnection of inflammation and angiogenesis was also demonstrated for substances able to reduce NFκB activation by VEGF and to inhibit the angiogenic cascade both *in vitro* and *in vivo* [36]. 

Our results showed that PL treatment of endothelial cells was able to stimulate both ERK1/2 and NFκB pathways. While both PL concentrations induced activation with the same early kinetics, the effect was different for later time-points, where only 20% PL induced a sustained activation status. To our knowledge, this is the first demonstration of NFκB activation in endothelial cells after PL treatment. However, some previous studies investigated PL effect on ERK1/2 and NFκB in cells involved in wound healing. A sustained activation of ERK1/2 had been demonstrated for endothelial cells when 20% PL was added to FBS-containing medium, showing that the effect of PL occurs through the stimulation of cell proliferation and migration, in a process strictly ERK1/2-dependent [23]. PL activation of ERK1/2 and NFκB pathways had been demonstrated in two separate studies in keratinocytes. In particular, the study by El Backly and coworkers [28] showed that 5% PL, approximately corresponding to a physiologic platelet concentration in the prelysate platelet suspension, exerted the highest effect on wound closure, associated with activation of NFκB. Both sub-physiologic (1%) and higher-than-physiologic (20%) concentrations resulted in a delaying effect, pointing to a dose-dependent effect of PL. As the PL used by these authors is more concentrated than the one we used (1x10^10^ vs. 1.3x10^9^ platelets/ml), their results are in accordance with our experiments. Another study showed only ERK1/2 involvement with no variation of NFκB phosphorylation in keratinocytes, whereas a slight but significant NFκB inactivation was observed in fibroblasts [37], a result that adds to demonstration of ERK1/2 activation in this cell type [12].

PL effect on different cells involved in wound repair supports this application for this platelet derivative; nevertheless, rapid leakage and short half-life of growth factors limits PL clinical application. Moreover, a chronic wound environment can induce premature growth factor degradation and inactivation caused by elevated levels of matrix metalloproteinase activity [38]. As PL efficacy is dependent on its availability to the injured tissue, a direct delivery at the wound site through controlled-release systems would enhance PL biological effects, protecting growth factors from enzymatic degradation and providing sustained delivery without loss of bioactivity. Recent studies evaluated this experimental approach. A PL-impregnated collagen/gelatin scaffold was investigated for wound repair, assessing PL optimal concentration [39]. PDGF-BB and TGF-β1 were released from the scaffold in a sustained manner after collagenase treatment. A 2x concentrated PL accelerated wound healing and enhanced cell proliferation and vessel growth, promoting the formation of dermis-like tissue. Another study concerned the development of sponge-like dressings, obtained by freeze-drying, based on chitosan glutamate and sodium hyaluronate for PL delivery to chronic skin wounds [40]. The effect of dressings loaded with PL on fibroblast proliferation and PDGF-AB release was compared with values observed for fresh PL, confirming dressing capability to maintain unaltered platelet growth factors.

## Conclusions

These data bring further scientific support to possible clinical application of platelet derivatives, suggesting the use of allogeneic PL for regenerative application. The use of PL for wound healing is supported by its effect on the activities of different cell types involved in this process. PL was able to substitute growth supplements such as FBS and growth factors, with a dose-related effect, with peculiar characteristics at the higher PL concentration evaluated. The use of a pool of healthy donors could minimize individual variability, representing also an advantage as compared to patients derivatives, encouraging application of allogeneic products. The development of controlled-release systems to protect growth factors and provide sustained delivery would enhance PL biological effects for clinical application in wound healing. 

## References

[1] Dulmovits BM, Herman IM (2012) Microvascular remodeling and wound healing: a role for pericytes. Int J Biochem Cell Biol 44: 1800-1812. doi:10.1016/j.biocel.2012.06.031. PubMed: 22750474. 22750474 PMC3455116

[2] Anitua E, Sánchez M, Nurden AT, Nurden P, Orive G et al. (2006) New insights into and novel applications for platelet-rich fibrin therapies. Trends Biotechnol 24: 227-234. doi:10.1016/j.tibtech.2006.02.010. PubMed: 16540193. 16540193

[3] Nurden AT (2011) Platelets, inflammation and tissue regeneration. Thromb Haemost 105 Suppl 1: S13-S33. doi:10.1160/THS10-11-0720. PubMed: 21479340. 21479340

[4] Radziwon-Balicka A, Moncada de la Rosa C, Jurasz P (2012) Platelet-associated angiogenesis regulating factors: a pharmacological perspective. Can J Physiol Pharmacol 90: 679-688. doi:10.1139/y2012-036. PubMed: 22512504. 22512504

[5] Ranzato E, Patrone M, Mazzucco L, Burlando B (2008) Platelet lysate stimulates wound repair of HaCaT keratinocytes. Br J Dermatol 159: 537-545. PubMed: 18616790. 18616790 10.1111/j.1365-2133.2008.08699.x

[6] Mazzucco L, Medici D, Serra M, Panizza R, Rivara G et al. (2004) The use of autologous platelet gel to treat difficult-to-heal wounds: a pilot study. Transfusion 44: 1013-1018. doi:10.1111/j.1537-2995.2004.03366.x. PubMed: 15225241. 15225241

[7] Borzini P, Mazzucco L (2005) Tissue regeneration and in loco administration of platelet derivatives: clinical outcome, heterogeneous products, and heterogeneity of the effector mechanisms. Transfusion 45: 1759-1767. doi:10.1111/j.1537-2995.2005.00600.x. PubMed: 16271101. 16271101

[8] Pawitan JA (2012) Platelet rich plasma in xeno-free stem cell culture: the impact of platelet count and processing method. Curr Stem Cell Res Ther 7: 329-335. doi:10.2174/157488812802481508. PubMed: 22849700. 22849700

[9] Reinisch A, Bartmann C, Rohde E, Schallmoser K, Bjelic-Radisic V et al. (2007) Humanized system to propagate cord blood-derived multipotent mesenchymal stromal cells for clinical application. Regen Med 2: 371-382. doi:10.2217/17460751.2.4.371. PubMed: 17635045. 17635045

[10] Bernardi M, Albiero E, Alghisi A, Chieregato K, Lievore C et al. (2013) Production of human platelet lysate by use of ultrasound for ex vivo expansion of human bone marrow-derived mesenchymal stromal cells. Cytotherapy 15: 920-929. doi:10.1016/j.jcyt.2013.01.219. PubMed: 23623274. 23623274

[11] Felice F, Lucchesi D, di Stefano R, Barsotti MC, Storti E et al. (2010) Oxidative stress in response to high glucose levels in endothelial cells and in endothelial progenitor cells Evidence for differential glutathione peroxidase-1 expression. Microvasc Res 80: 332-338. doi:10.1016/j.mvr.2010.05.004. PubMed: 20471990. 20471990

[12] Ranzato E, Mazzucco L, Patrone M, Burlando B (2009) Platelet lysate promotes in vitro wound scratch closure of human dermal fibroblasts: different roles of cell calcium, P38, ERK and PI3K/AKT. J Cell Mol Med 13: 2030-2038. doi:10.1111/j.1582-4934.2008.00467.x. PubMed: 19267882. 19267882 PMC6512364

[13] Da Pozzo E, Barsotti MC, Bendinelli S, Martelli A, Calderone V et al. (2012) Differential Effects of Fondaparinux and Bemiparin on Angiogenic and Vasculogenesis-like processes. Thromb Res 130: e113-e122. doi:10.1016/j.thromres.2012.03.013. PubMed: 22497885. 22497885

[14] Papanas N, Eleftheriadou I, Tentolouris N, Maltezos E (2012) Advances in the topical treatment of diabetic foot ulcers. Curr Diabetes Rev 8: 209-218. doi:10.2174/157339912800563963. PubMed: 22429013. 22429013

[15] Occleston NL, O'Kane S, Laverty HG, Cooper M, Fairlamb D et al. (2011) Discovery and development of avotermin (recombinant human transforming growth factor beta 3): a new class of prophylactic therapeutic for the improvement of scarring. Wound Repair Regen 19 Suppl 1: s38-s48. doi:10.1111/j.1524-475X.2011.00711.x. PubMed: 21793965. 21793965

[16] Doucet C, Ernou I, Zhang Y, Llense JR, Begot L et al. (2005) Platelet lysates promote mesenchymal stem cell expansion: a safety substitute for animal serum in cell-based therapy applications. J Cell Physiol 205: 228-236. doi:10.1002/jcp.20391. PubMed: 15887229. 15887229

[17] Mazzucco L, Balbo V, Guaschino R (2012) "Reasonable compromise" to define the quality standards of platelet concentrate for non-transfusion use (CPunT). Transfus Apher Sci 47: 207-211. doi:10.1016/j.transci.2012.06.006. PubMed: 22795794. 22795794

[18] Jonsdottir-Buch SM, Lieder R, Sigurjonsson OE (2013) Platelet lysates produced from expired platelet concentrates support growth and osteogenic differentiation of mesenchymal stem cells. PLOS ONE 8: e68984. doi:10.1371/journal.pone.0068984. PubMed: 23874839. 23874839 PMC3708923

[19] Novosel EC, Kleinhans C, Kluger PJ (2011) Vascularization is the key challenge in tissue engineering. Adv Drug Deliv Rev 63: 300-311. doi:10.1016/j.addr.2011.03.004. PubMed: 21396416. 21396416

[20] Yang DH, Hsu CF, Lin CY, Guo JY, Yu WC et al. (2013) Krüppel-like factor 10 upregulates the expression of cyclooxygenase 1 and further modulates angiogenesis in endothelial cell and platelet aggregation in gene-deficient mice. Int J Biochem Cell Biol 45: 419-428. doi:10.1016/j.biocel.2012.11.007. PubMed: 23178857. 23178857

[21] Kajdaniuk D, Marek B, Foltyn W, Kos-Kudła B (2011) Vascular endothelial growth factor (VEGF) - part 1: in physiology and pathophysiology. Endokrynol Pol 62: 444-455. PubMed: 22069106. 22069106

[22] Fekete N, Gadelorge M, Fürst D, Maurer C, Dausend J et al. (2012) Platelet lysate from whole blood-derived pooled platelet concentrates and apheresis-derived platelet concentrates for the isolation and expansion of human bone marrow mesenchymal stromal cells: production process, content and identification of active components. Cytotherapy 14: 540-554. doi:10.3109/14653249.2012.655420. PubMed: 22296115. 22296115 PMC3400099

[23] Ranzato E, Boccafoschi F, Mazzucco L, Patrone M, Burlando B (2010) Role of ERK1/2 in platelet lysate-driven endothelial cell repair. J Cell Biochem 110: 783-793. doi:10.1002/jcb.22591. PubMed: 20512938. 20512938

[24] Radziwon-Balicka A, Moncada de la Rosa C, Zielnik B, Doroszko A, Jurasz P (2013) Temporal and pharmacological characterization of angiostatin release and generation by human platelets: implications for endothelial cell migration. PLOS ONE 8: e59281. doi:10.1371/journal.pone.0059281. PubMed: 23555012. 23555012 PMC3598756

[25] Sum R, Hager S, Pietramaggiori G, Orgill DP, Dee J et al. (2007) Wound-healing properties of trehalose-stabilized freeze-dried outdated platelets. Transfusion 47: 672-679. doi:10.1111/j.1537-2995.2007.01170.x. PubMed: 17381626. 17381626

[26] Battinelli EM, Markens BA, Italiano JE (2011) Release of angiogenesis regulatory proteins from platelet alpha granules: modulation of physiologic and pathologic angiogenesis. Blood 118: 1359-1369. doi:10.1182/blood-2011-02-334524. PubMed: 21680800. 21680800 PMC3152500

[27] Moncada de la Rosa C, Radziwon-Balicka A, El-Sikhry H, Seubert J, Ruvolo PP et al. (2013) Pharmacologic protein kinase Cα inhibition uncouples human platelet-stimulated angiogenesis from collagen-induced aggregation. J Pharmacol Exp Ther 345: 15-24. doi:10.1124/jpet.112.200881. PubMed: 23386249. 23386249

[28] El Backly R, Ulivi V, Tonachini L, Cancedda R, Descalzi F et al. (2011) Platelet lysate induces in vitro wound healing of human keratinocytes associated with a strong proinflammatory response. Tissue Eng Part A 17: 1787-1800. doi:10.1089/ten.tea.2010.0729. PubMed: 21385008. 21385008

[29] Burnouf T, Chou ML, Wu YW, Su CY, Lee LW (2013) Antimicrobial activity of platelet (PLT)-poor plasma, PLT-rich plasma, PLT gel, and solvent/detergent-treated PLT lysate biomaterials against wound bacteria. Transfusion 53: 138-146. doi:10.1111/j.1537-2995.2012.03668.x. PubMed: 22563709. 22563709

[30] Roskoski R (2012) ERK1/2 MAP kinases: structure, function, and regulation. Pharmacol Res 66: 105-143. doi:10.1016/j.phrs.2012.04.005. PubMed: 22569528. 22569528

[31] Lee SH, Zahoor M, Hwang JK, Min, Choi KY (2012) Valproic acid induces cutaneous wound healing in vivo and enhances keratinocyte motility. PLOS ONE 7: e48791. doi:10.1371/journal.pone.0048791. PubMed: 23144972. 23144972 PMC3492241

[32] Watson A, Morris VL, Chan BM (2009) Coordinated integrin and growth factor regulation of primary keratinocyte migration mediated through extracellular signal regulated kinase and phosphoinositide 3-kinase. Arch Dermatol Res 301: 307-317. doi:10.1007/s00403-009-0945-7. PubMed: 19330341. 19330341

[33] Lan W, Petznick A, Heryati S, Rifada M, Tong L (2012) Nuclear Factor-κB: central regulator in ocular surface inflammation and diseases. Ocul Surf 10: 137-148. doi:10.1016/j.jtos.2012.04.001. PubMed: 22814642. 22814642

[34] Melchionna R, Bellavia G, Romani M, Straino S, Germani A et al. (2012) C/EBPγ regulates wound repair and EGF receptor signaling. J Invest Dermatol 132: 1908-1917. doi:10.1038/jid.2012.51. PubMed: 22437320. 22437320

[35] Valacchi G, Pecorelli A, Sticozzi C, Torricelli C, Muscettola M et al. (2011) Rottlerin exhibits antiangiogenic effects in vitro. Chem Biol Drug Des 77: 460-470. doi:10.1111/j.1747-0285.2011.01121.x. PubMed: 21435184. 21435184

[36] Kim YW, West XZ, Byzova TV (2013) Inflammation and oxidative stress in angiogenesis and vascular disease. J Mol Med (Berl) 91: 323-328. doi:10.1007/s00109-013-1007-3. PubMed: 23430240. 23430240 PMC3656485

[37] Ranzato E, Martinotti S, Volante A, Mazzucco L, Burlando B (2011) Platelet lysate modulates MMP-2 and MMP-9 expression, matrix deposition and cell-to-matrix adhesion in keratinocytes and fibroblasts. Exp Dermatol 20: 308-313. doi:10.1111/j.1600-0625.2010.01173.x. PubMed: 20955204. 20955204

[38] Losi P, Briganti E, Errico C, Lisella A, Sanguinetti E et al. (2013) Fibrin-based scaffold incorporating VEGF- and bFGF-loaded nanoparticles stimulates wound healing in diabetic mice. Acta Biomater 9: 7814-7821. doi:10.1016/j.actbio.2013.04.019. PubMed: 23603001. 23603001

[39] Ito R, Morimoto N, Pham LH, Taira T, Kawai K et al. (2013) Efficacy of the controlled release of concentrated platelet lysate from a collagen/gelatin scaffold for dermis-like tissue regeneration. Tissue Eng Part A 19: 1398-1405. doi:10.1089/ten.tea.2012.0375. PubMed: 23427847. 23427847

[40] Rossi S, Faccendini A, Bonferoni MC, Ferrari F, Sandri G et al. (2013) "Sponge-like" dressings based on biopolymers for the delivery of platelet lysate to skin chronic wounds. Int J Pharm 440: 207-215. doi:10.1016/j.ijpharm.2012.07.056. PubMed: 22884830. 22884830

