# Correction: Effect of Platelet Lysate on Human Cells Involved in Different Phases of Wound Healing

**DOI:** 10.1371/journal.pone.0093352

**Published:** 2014-03-19

**Authors:** The PLOS ONE Staff

The name of the first author is incorrectly represented in the Citation. The Citation should read: Barsotti MC, Losi P, Briganti E, Sanguinetti E, Magera A, et al. (2013) Effect of Platelet Lysate on Human Cells Involved in Different Phases of Wound Healing. PLoS ONE 8(12):

e84753. doi:10.1371/journal.pone.0084753.

## References

[1] Chiara Barsotti M, Losi P, Briganti E, Sanguinetti E, Magera A, et al (2013) Effect of Platelet Lysate on Human Cells Involved in Different Phases of Wound Healing. PLoS ONE 8(12): e84753 doi:10.1371/journal.pone.0084753 24386412 10.1371/journal.pone.0084753 PMC3873992

